# Assessment of Heavy Metals in Municipal Sewage Sludge: A Case Study of Limpopo Province, South Africa

**DOI:** 10.3390/ijerph110302569

**Published:** 2014-03-03

**Authors:** Kudakwashe K. Shamuyarira, Jabulani R. Gumbo

**Affiliations:** Department of Hydrology and Water Resources; University of Venda, P/Bag x5050, Thohoyandou 0950, South Africa

**Keywords:** heavy metals, sewage sludge, effluent, silver

## Abstract

Heavy metals in high concentrations can cause health and environmental damage. Nanosilver is an emerging heavy metal which has a bright future of use in many applications. Here we report on the levels of silver and other heavy metals in municipal sewage sludge. Five towns in Limpopo province of South Africa were selected and the sludge from their wastewater treatment plants (WWTPs) was collected and analysed. The acid digested sewage sludge samples were analysed using Inductively Coupled Plasma-Optical Emission Spectroscopy (ICP-OES) and Inductively Coupled Plasma-Mass Spectrometry (ICP-MS) methods. The concentrations of silver found were low, but significant, in the range 0.22 to 21.93 mg/kg dry mass. The highest concentration of silver was found in Louis Trichardt town with a concentration of 21.93 ± 0.38 mg/kg dry mass while the lowest was Thohoyandou with a concentration of 6.13 ± 0.12 mg/kg dry mass. A control sludge sample from a pit latrine had trace levels of silver at 0.22 ± 0.01 mg/kg dry mass. The result showed that silver was indeed present in the wastewater sewage sludge and at present there is no DWAF guideline standard. The average Cd concentration was 3.10 mg/kg dry mass for Polokwane municipality. Polokwane and Louis Trichardt municipalities exhibited high levels of Pb, in excess DWAF guidelines, in sludge at 102.83 and 171.87 mg/kg respectfully. In all the WWTPs the zinc and copper concentrations were in excess of DWAF guidelines. The presence of heavy metals in the sewage sludge in excess of DWAF guidelines presents environmental hazards should the sludge be applied as a soil ameliorant.

## 1. Introduction

Technological advancement, increase in incomes and betterment of standards of living have resulted in the increased demand for almost everything. With more income people use more resources and have more wants and needs [1]. Such a situation has led to an increase in the number of uses of heavy metals. Yearly more and more amounts of heavy metals are being used and incorporated into products. These heavy metals are toxic to human health and the environment at trace level concentrations, which is a major cause of concern [2]. 

Nanotechnology is an emerging technology with a sparkling bright future, and as a result the use of nanoparticles has risen over many decades. Over the past two decades the use has risen even more dramatically due to technological advancements [3,4]. Nanoparticles are natural or man-made particles with at least two dimensions between 1 and 100 nanometers [5]. Nanosilver in particular is one of the most frequently used elements in many applications and it is of particular interest because it is a heavy metal yet it also has a nanoparticle nature. Nanoparticles are very minute particles which have more anthropogenic origin than natural origin. Due to their size they find a vast number of uses in food processing, textile production, cosmetics, medical devices and many other fields. Due to the widespread use in consumer products, it is likely that nanoparticles are entering water streams and the treatment facilities that process wastewater [3]. 

Emerging technologies have always posed hazards to the people and environment. Many diverse nanoparticles are being engineered and are formed in daily applications [5]. Due to the size and morphology, nanoparticles have very different properties than the original bulk material [6]. Thus the properties of bulk silver have been well documented and its effects in various forms are well known. This has allowed even the formulation of safety procedures and standards. As for nanosilver, much of the effects are potential or just proposed. According to El-Badawy *et al.* [7] bulk silver was first used around 3,000 B.C and nanosilver was first used just over 100 years ago. It is clear that nanosilver use is very new when compared to bulk silver use. Hence there is lack of knowledge with regards to nanosilver [8,9]. Due to the nature of nanosilver uses it may find its way into wastewater treatment plants and sludge according Benn and Westerhoff [10]. Sludge can be used as a soil ameliorant, that is, for improving the state of the soil [11]. Sludge can also be disposed of into landfills or it can be incinerated. Land application of sludge is a practice which can lead to the contamination of water and soil by heavy metals and nanosilver. To deal with something one has to quantify and characterize it. Thus possible hazards and solutions can only be deduced after knowing the occurrence and amount of heavy metals, including nanosilver, entering our sludge.

Nanoparticles are considered emerging contaminants. NIWA [12] describes emerging contaminants as chemicals which are not commonly monitored but have the potential to cause adverse effects to the environment and humans. They are of major concern as history has shown that emerging contaminants can cause catastrophic effects because little or no attention is paid to their nature and effects in their infancy. As noted by NIOSH [13], risk assessment of nanoparticles has been limited due to lack of information. This study assessed the availability and presence of heavy metals in the sewage sludge of selected municipalities in Limpopo Province. The specific objectives were to detect the presence of silver; to determine the concentration of silver in the sewage sludge and to determine the levels of heavy metals in the sewage sludge.

## 2. The Study Area

**Figure 1:**
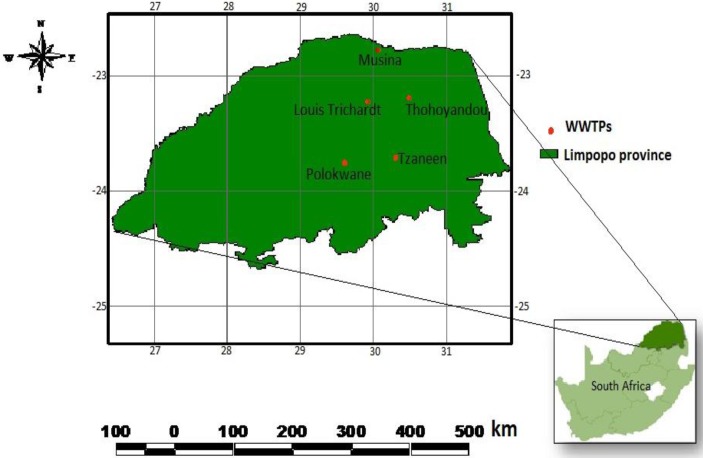
The location of municipal wastewater treatment plants.

Limpopo Province is located on the northern part of South Africa. The five wastewater treatment plants in Limpopo were randomly selected (Figure 1).

Limpopo Province is one of nine provinces of South Africa and the total human population of Limpopo province is approximately 5.6 million and the majority live in rural areas [14]. In rural areas of South Africa, pit latrines are the major sanitary facilities [14,15]. Stats SA [14] found that on average there were 19.7% of Limpopo households that were using flush toilets that were connected to the sewage system. 

## 3. Materials and Methods

### 3.1. Sample Sites and Sample Collection

There are 62 Municipal Wastewater Treatment plants in the Limpopo province as of 2009 [16]. The five WWTPs were selected based on the production of sludge as part of their wastewater treatment process and in line with the survey by EPA [17]. This criterion resulted in elimination of other WWTPs which did not produce sludge, as is the case of Mutale WWTP. A dried sludge sample from a pit latrine was also analysed for comparison purposes.

With the formal permission of Limpopo local municipalities, samples of dried sludge were collected from the five WWTPs. The grab samples were collected in November 2012 from the sludge drying beds. For each of the WWTPs, a representative sample was taken comprising dry sludge from different beds**.** Each sample was collected in a plastic bag, labeled and then placed in a cooler box with ice and after transport they were placed in a refrigerator at 4 °C until further analysis.

### 3.2. Preparation of Samples

Samples were subjected to nitric acid digestion according to the EPA guidelines [18,19]. The following procedure used was used:

- 2 g (or 0.002 kg) of the milled sewage sludge sample was weighed and placed in a conical flask.
- 20 mL of HNO_3_ (55% concentration) were added
- Heated at 90 °C for 45 minutes.
- Temperature was the increased to 150 °C for 10 min.
- During heating and boiling, 10 mL of HNO_3_ (55% concentration) was added periodically three times to make sure that the liquid remains.
- The mixture was allowed to cool at room conditions.
- Following cooling, the samples were filtered into 100 mL volumetric flasks and filled to the mark with distilled water.

### 3.3. Metal Analysis by ICP AES and ICP-MS

The digested samples were filtred through 0.45 µm membrane filter (GVS Filter, Indiapolis, IN, USA) into 15 mL centrifuge tubes and sent for analysis at Stellenbosch University in duplicate by ICP-AES (Thermo ICAP 6300 instrument, Thermo Electron Limited, Cambridge, UK) and ICP-MS (Agilent 7700 instrument, Agilent Technologies Inc., Tokyo, Japan). The Thermo ICAP 6300 instrument reported the elements in parts per million (ppm) concentrations. The instrument is able to measure more than 40 elements at a detection limit of 0.01 ppm. ICP-MS was the employed to analyses for the trace elements which are found at very low concentrations. The Agilent 7700 instrument reported the trace element concentrations in parts per billion (ppb) this instrument is able to measure trace and ultra-trace element concentrations even down to parts per trillion (ppt). Both instruments were calibrated using the US EPA method 6020A [18].

### 3.4. Data Analysis

The data acquisition and processing was controlled by ICP-AES and ICP-MS software. The results were expressed as mg per kg as indicated by the following equation, with weight of sludge expressed in kg:

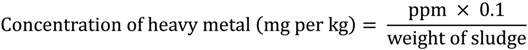


MS Excel was used calculation of average, standard deviation and drawing of graphs. 

## 4. Results and Discussion

### 4.1. Presence of Silver in the Sludge from Limpopo Province

**Figure 2:**
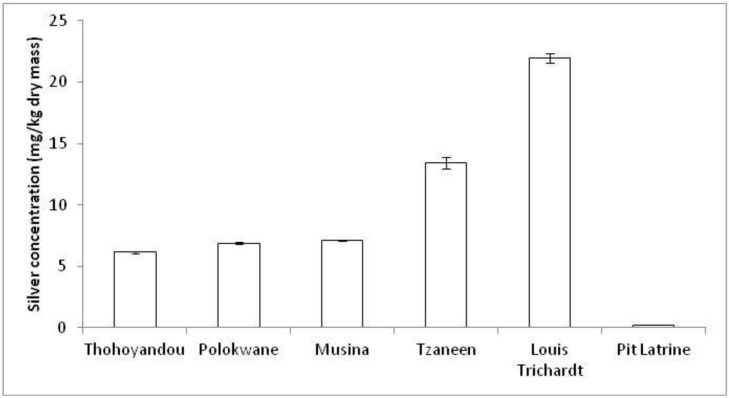
Concentrations of silver (mg/kg dry mass) in sludge for each municipality.

In all the samples that were analysed using ICP-MS, silver (Ag) was found to be present and the concentrations showed variations among the sewage plants (Figure 2). Our research results were however within range and comparable to the results of Kim *et al.* [3] that were done in the USA. They analysed the sewage sludge of 74 WWTPs and they found a range of 1.94 to 856 mg/kg dry mass (d. m.). The range of our results was from 0.22 to 21.93 mg/ kg d. m.

**Figure 3:**
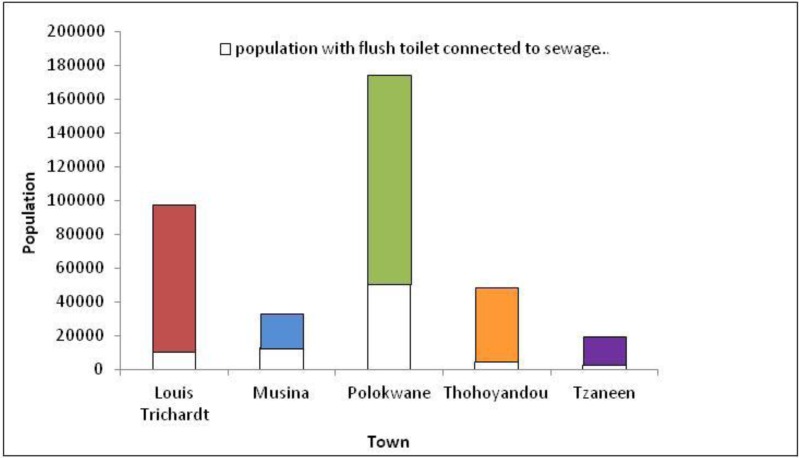
Population with flush toilet connected to sewage system for each town [14].

The towns of Louis Trichardt and Tzaneen had the highest Ag concentrations of 21.93 ± 0.38 mg/kg d. m. and 13.40 ± 0.46 mg/kg d. m., respectively, while the lowest was Thohoyandou with a concentration of 6.13 ± 0.12 mg/kg d. m. of silver (Figure 2). This suggests that residents in Louis Trichardt probably use more of the silver-containing cosmetics than the rest of the towns. The pit latrine sample had trace silver concentration of 0.22 ± 0.01 mg/kg d. m. and was the lowest since households using pit latrines do not usually dispose their wastewater into the pit latrine. Instead, they pour their bathwater and laundry water usually on compost or water the trees [20]. The result of 6.89 ± 0.11 mg/kg d. m. observed in Polokwane is unexpected, as it has a larger population than the other towns. The populations of Polokwane, Louis Trichardt, Thohoyandou, Musina and Tzaneen according to Stats SA [14], are shown in Figure 3. 

The Polokwane municipality has the highest number of flush toilet users connected to the sewage system (Figure 3). Tzaneen municipality also had an unexpectedly high silver concentration, but the smallest population of all the towns. This could explain why Tzaneen, a tourist town, had the second highest silver concentration. In Sweden, the Swedish Environmental Protection Agency (SEPA) has recommended a silver guideline value of 8 mg/kg in sludge destined for agricultural applications [2], thus the towns of Louis Trichardt and Tzaneen had silver concentration in excess of the SEPA guidelines.

Please note the presence of silver in the sewage sludge was assumed to originate from the use of silver-containing nanoproducts such as Samsung washing machines, Nivea Silver Protect, Nivea Silver Spray and Clicks Silver Assorted Sheer Plasters. The survey of Modika [21] showed that these silver nanoparticle consumer products were available in the stores and pharmacies in Thohoyandou and Louis Trichardt and also that people were buying and using these consumer goods in the study area. The sewage sludge from the pit latrine was found to contain trace levels of silver. Although the concentration was low it supported the assumption that silver was coming from domestic use of - containing cosmetics and equipment. 

To date there are no DWA regulations or guidelines in the levels of silver in sewage sludge in South Africa. However the dried sludge may be applied to land as a soil conditioner in South Africa [11]. In the USA, the EPA also has not regulated the levels of silver in sludge in light of emerging silver nanoproducts, but has regulated silver ion generators as pesticides under the Federal Insecticide, Fungicide, and Rodenticide Act (FIFRA) [9,22]. In the Europe Union (EU), silver has not been listed among the 33 “priority hazardous pollutants” but the EU biosolid directive (sewage sludge directive 86/278/EEC) requires that all biocides including silver to be screened and approved by 14 May 2014 [2].

### 4.2. The Presence of Heavy Metals in Municipal Sludge

The results show that the heavy metals, Cr, Ni, Cd, Pb, Cu and Zn, were present in the sewage sludge and their concentrations were variable (Table 1). 

**Table 1:** The heavy metals in municipal sewage sludge (mg/kg dry mass).

Metal	Thohoyandou	Polokwane	Tzaneen	Louis Trichardt	Musina	Pit toilet	DWAF guidelines * [10]	
							Total maximum threshold	Maximum permissible level
**Cr ^#^**	64.36	134.48	53.47	97.12	35.07	44.13	350	450
**Ni**	33.90	47.27	31.34	51.43	35.23	18.89	150	200
**Cd**	0.82	3.10 **	1.39	1.66	1.06	0.32	3	5
**Pb**	34.56	102.83 **	52.26	171.87 **	21.28	17.96	100	150
**Cu**	377.95 **	324.83 **	263.68 **	499.28 **	626.00 **	80.80	120	375
**Zn**	1192.50 **	1551.50 **	951.25 **	1732.00 **	1031.75 **	303.83 **	200	700

Notes: ^#^ Total chromium; * DWAF guidelines for metal limit receiving high sludge loads. **Exceed DWAF guidelines.

The sludge may be of beneficial use such as landfill cover and once-off high rate application to exposed mine tailings. However if the sludge was to be applied for landfill cover, the level of heavy metals in the sludge greatly exceed the DWAF guidelines [11]. Thus for once-off high rate application of sludge to land, some of the heavy metals complied with DWAF guidelines with exception of Cd, Pb, Cu and Zn which exceeded the DWAF guidelines (Table 1). The following heavy metals: Cd, Pb, Cu and Zn, were in excess of the DWA guidelines and these metals have a significant environmental impact and are hazardous to human health in the long term.

#### 4.2.1. Cadmium

The average cadmium (Cd) concentration was 3.10 ± 0.16 mg/kg d. m. for Polokwane municipality, in excess of the DWAF guideline for total maximum threshold, but lower than the maximum permissible level (Table 1). This was higher in comparison with the study of Morrison *et al.* [2] who found an average Cd of 1.9 mg/kg d. m. for Shornville upper WWTP, Eastern Cape, South Africa. A major source of cadmium was probably the use of rubber tires and heavy traffic in the capital city of Polokwane [2,23]. This can be supported also by the fact that Polokwane WWTP is located close to high traffic roads from which cadmium may have entered the stormwater drains that are connected to the sewage plant. 

#### 4.2.2. Lead

The lead (Pb) concentrations were in the range 21.3 to 171.85 mg/kg d. m., but high Pb concentrations in excess of DWAF guideline values, were found in Polokwane and Louis Trichardt (Table 1). These research results were comparable to the findings of Morrison *et al.* [2] in which they found Pb values in the range of 69 to 365 mg/kg d. m. and an EPA [17] survey which found Pb in the range of 5.81 to 540 mg/kg d. m. A major source of Pb was probably the use of leaded petrol. With the introduction and the preference of unleaded petrol in the past decade, leaded petrol has been phased out. The lower Pb concentrations in this study could be due to the phasing out of leaded petrol in South Africa [24,25] in comparison to the year 2002 when Morrison *et al.* [2] conducted their study. This can be supported also by the fact that Louis Trichardt and Polokwane WWTPs are located close to high traffic roads from which Pb may have entered the stormwater drains that are connected to the sewage plants. A possible reason for the lower Musina and Thohoyandou concentrations could be that the stormwater drains are not connected to the sewage plants.

#### 4.2.3. Copper

**Figure 4:**
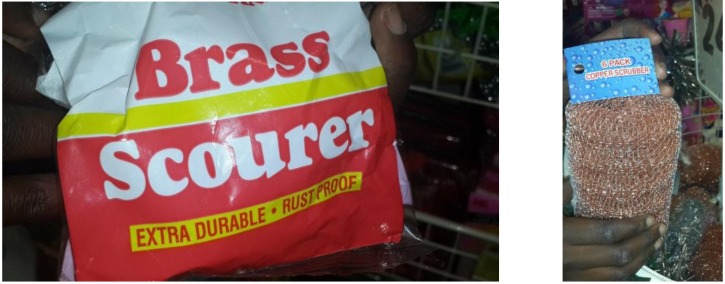
Cleaning materials containing copper and zinc.

The concentrations of copper (Cu) from all the study towns were very high, and above the DWAF guideline values (Table 1). The Cu concentrations were in the range 263.68 to 626.00 mg/kg d. m. The Musina municipality had the highest concentration of Cu. The reason for the high Cu concentrations could have been caused probable by the corrosion of the water supply pipes, whose composition includes copper. This was supported by the study of Tjandraatmadja *et al.* [26] who found that about 46% of the copper in sewage sludge came from the water supply connected to the household pipes in Melbourne, Australia. Musina also has abandoned copper mines and the leachate from these could have got into the raw water used for the supply of the town [27]. Our research results were comparable to the findings of Morrison *et al.* [2] in which they found Cu values in the range of 245 to 441 mg/kg d. m. and EPA [17] survey which found Cu in the range of 115 to 2,580 mg/kg d. m. An explanation for the high levels of Cu in sludge may be the use of brass (contains copper and zinc) and copper scrubbers as abrasives in general cleaning in household kitchens and the washing of pots (Figure 4). 

#### 4.2.4. Zinc

The concentrations of zinc (Zn) from all the study towns were very high and above the DWAF guideline values (Table 1). The Zn concentrations were in the range 951.25 to 1,732.00 mg/kg d. m. and were comparable to the studies of Morrison *et al.* [2] and EPA [17] in the ranges of 1,600 to 4,100 mg/kg d. m. and 216 to 8,550 mg/kg d. m. respectfully. The origin of Zn in the sludge may be due to the use of brass as a household cleaning material (Figure 4). Secondly Zn is a constituent of galvanized steel including water distribution pipes and the possibility is that its presence might be due to corrosion and the metal may find its way to the WWTPs [28]. 

## 5. Conclusions

The study showed that indeed silver was present in the sewage sludge of the five selected municipalities and in a pit latrine sludge. The observed silver concentrations most likely contain nanosilver, although further characterization is needed to assess whether the silver is in nanoparticle form or not. The concentrations of silver in the municipal sewage sludge were in the range of 6.13 to 21.93 mg/kg of dry mass, while the pit latrine (control) exhibited trace silver levels of 0.22 mg/kg dry mass. At present in South Africa there are no regulations to the concentration of silver in sewage sludge. Four municipalities had average Cd concentrations below the DWAF total maximum threshold, with the exception of Polokwane, with an average Cd concentration of 3.10 mg/kg dry mass in excess of DWAF guideline for total maximum threshold, but lower than the maximum permissible level. Polokwane and Louis Trichardt municipalities exhibited high levels of Pb in sludge, in excess of DWAF guidelines, at 102.83 and 171.87 mg/kg dry mass respectively. In all the WWTPs the zinc and copper concentrations were in excess of DWAF guidelines. The presence of heavy metals including silver may be hazardous to the environment and affect human health should the sludge be applied to agricultural fields in light of the potential of the heavy metals accumulate in the fields.
